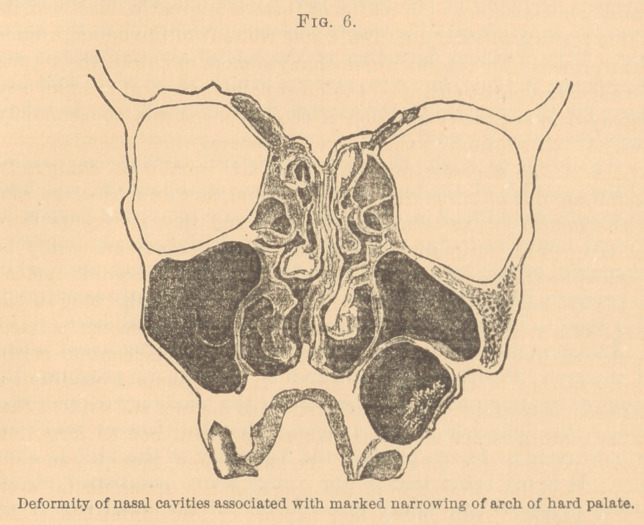# The Influence of Adenoid Hypertrophy at the Vault of the Pharynx upon the Development of the Hard Palate

**Published:** 1891-01

**Authors:** D. Bryson Delavan

**Affiliations:** New York


### International Dental Journal.

Vol. XII. January, 1891. No. 1.

### Original Communications.[1]

### THE INFLUENCE OF ADENOID HYPERTROPHY AT THE VAULT OF THE PHARYNX UPON THE DEVELOPMENT OF THE HARD PALATE.[2]

BY DR. D. BRYSON DELAVAN, NEW YORK.

In no department of the invaluable art here represented have greater advances been made of late than in that which has for its object the correction of deformities resulting from faulty positions of the permanent teeth. The intelligent study of unusual conditions, the adaptation of mechanical appliances of extraordinary ingenuity, and, above all, the accurate appreciation of the possibilities present in a given case, all of these factors have contributed to produce results which, but a few years ago, would have been considered impracticable. Surely to the relief of the malformations to be discussed in this paper dental surgery has amply contributed its share. In the face of what may be considered a veritable triumph, however, we are confronted with the fact that, in a large number of the worst cases which apply for treatment, there are present in the upper jaw certain deformities of the bony arch itself, which, despite the utmost success in the restoration of the teeth to

---

[1] The editor and publishers are not responsible for the views of authors of papers published in this department, nor for any claim to novelty, or otherwise, that may be made by them. No papers will be received for this department that have appeared in any other journal published in the country.

[2] Read before the New York Odontological Society, November 16, 1890.

their normal line, fail to disappear in the presence of the otherwise improved conditions, and persist throughout the remainder of the patient's life.

It is my purpose to explain somewhat minutely the exact nature of these deformities, and to call your attention to a condition which, I believe, is their most common and efficient cause,—namely, to the disease known under the name of "adenoid hypertrophy at the vault of the pharynx."

High above the soft palate, and posterior to the nasal cavities, is that part of the pharynx which forms its roof or vault. (*Vide* Figs. 1 and 2.) Normally, this should be a free, empty space,

Fig. 1.

The upper pharynx in the healthy condition; the vault or arched roof, *A*, being well rounded and freefrom obstructing tissue, and the entrances to the nose, *C*, being entirely clear and open. (*Lefferts.*)

arched from before backward, and from side to side. This region is covered with mucous membrane and it contains a small amount of adenoid tissue, the same structure as that of which the faucial tonsils are mainly composed. Like the faucial tonsils, this pharyngeal tonsil, as it is sometimes called, is susceptible of chronic enlargement, or hypertrophy, and in this condition of chronic enlargement it constitutes the disease which forms the subject of our discussion. It is not necessary that an elaborate description of this adenoid hypertrophy at the vault of the pharynx be given here, as that has been done many years ago by the distinguished Professor Wilhelm Meyer, of Copenhagen, in his classic paper on the subject. It must be understood, however, that adenoid hypertrophy involves a filling up of that part of the respiratory channel which lies behind the nasal cavities, on the one hand, and above the soft palate, on the other, by a tissue closely resembling, if not

FIG. 2.

Side view of the pharynx, representing the same normal condition as Fig, 1. In natural respiration the mouth is closed and the current of air enters through the nose, where it is first filtered, warmed, and furnished with moisture, and then passed directly downward through the larynx and trachea into the lungs.

identical with, that which composes the tonsils. (*Vide* Figs. 3 and 4.) Not only is the cavity of the upper pharynx or post-

FIG. 3.

The upper pharynx, showing a large adenoid growth, *A*, springing from the vault, filling the superior part of the pharynx, covering the tube leading to the ear, *C*, and obstructing the entrances to the nose, *B*. (*Lefferts.*)

nasal space more or less filled by the actual mechanical presence of the growth itself, but it is also obstructed by the excretion of large

Fig. 4.

Side view of Fig. 3, showing obstruction, *A*, in upper part of pharynx. Owing to this the patient must breathe through the mouth.

quantities of viscid mucus, which still further tend to occlude the passage and to interfere with nasal respiration. Thus it is made more or less difficult, or even impossible, for the patient to respire through the nose, and he becomes, as it is commonly called, a "mouth-breather." Now, the habit of mouth-breathing is most pernicious. This proposition will be evident when we call to mind the exceedingly important *rôle* played by the nose in the process of respiration, its physiological action being to free the inspired air from dust, to raise its temperature, and to furnish it with a large amount of moisture. Moreover, in the mouth-breather, it is not alone the quality of the air which is unnatural, but, what is even of greater importance, the actual amount received into the lungs is diminished, so that the child fails to secure the necessary supply of oxygen. It is of the utmost importance to the whole physical economy that the respiratory function of the nose be normally performed. The point which is of chief interest to us is that there are no parts of the body which suffer greater ills from mouth-breathing than do the nose itself and the bony structures in immediate relation with it. This statement has been amply proved by experiments upon animals (Ziem), for it has been found that if one nostril of a young rabbit be permanently occluded, the creature allowed to attain its full growth, and then killed and dissected, not only will the nasal cavity of the affected side be under-developed and deformed, but the whole of that side of the face will be asymmetrical

Fig. 5.

Asymmetry of nasal cavities.

and distorted. This, we believe, is equally true in the human being, for it is a matter of common observation to find deformities corresponding to the above in individuals who have suffered occlusion of one nasal cavity, such as may arise from a partial filling of the upper pharynx or from hypertrophy of the nasal mucous membrane. If it be true that occlusion of one nasal cavity will result in unilateral deformity of the neighboring parts, it is likewise possible that entire stoppage of nasal respiration through the occlusion of both nostrils will affect seriously the development of both sides of the face, and that we shall find, in consequence, deformities of its skeleton, which may exert an important influence upon parts with which the nose is not immediately connected.

Clinically, such cases are constantly seen; and in children who have been mouth-breathers from an early age, we find that, associated with deformities of the nose, there exist changes in the sinuses adjacent to the nose, by reason of which the whole shape of the face may be altered. What is of greater interest to us, alterations in the shape of the hard palate occur, both in its horizontal and its transverse diameters. By reason of these it is greatly narrowed, its roof is thrown upward, and the whole contour of the dental arch is transformed. To describe these changes more minutely, the results of adenoid hypertrophy upon the hard palate are most prominently displayed in the faulty position of the upper incisors, so commonly noticed. In addition to this, there are several other attendant conditions of the superior maxilla which are worthy of study. In examining one of these cases, we shall find, as a rule, that the roof of the mouth is exceedingly narrowed, and that it recedes from below upward until, sometimes, its highest point can hardly be touched by the tip of the patient's tongue. In other words, instead of the transverse diameter of the roof of the mouth being in the shape of a well-rounded, semicircular arch, it has become pointed, or shaped like an inverted letter Λ. Normally the contour of the part was that of the Norman arch. In its changed condition it has become Gothic. This peculiarity is so distinct and evident that it can hardly escape notice, and once seen will not be forgotten. Accompanying this deformity of the hard palate, there is generally a corresponding alteration in the shape of the upper dental arch, the transverse diameter of which is more or less diminished. This is particularly the case anteriorly, and is usually most pronounced in that part of the arch which lies forward of the canines. The result of this is to change the shape of the dental arch in a way analogous to that described above as characteristic of the hard palate,—namely, from the rounded to the V-shaped contour,—so that the normal curvature of the arch is entirely altered. Chatellier suggests that this condition, constituting the so-called "prognatism," should be considered less a race-peculiarity than a true pathological state. The prominence of the anterior region of the alveolar arch is still further increased by the projection forward of the superior maxilla at this point, and of the upper front teeth. These latter often project so considerably as to push forward the upper lip, under which they appear from below. The more the dental arch has become narrowed, the more prominent is this part of the superior maxillary bone likely to be.

In examining one of these cases, it will be found that the walls of the nasal cavities and the roof of the mouth have not kept pace with the general development of the head, but that they are, in many instances, smaller. On the other hand, the alveoli of the superior maxilla, being subjected to the physiological stimulation of functional activity, rapidly develop up to full maturity. The expression of the stunting of growth of the vomer, the septum of the

FIG. 6.

Deformity of nasal cavities associated with marked narrowing of arch of hard palate.

nose and the sinuses, is seen along the line of union of the septum with the hard palate, which is more or less fixed centrally, while the alveoli continue to grow and increase downward (Spicer).

It is certainly suggestive to remember that among primitive races, such as the Indian and the negro, in whom mouth-breathing is almost unknown, these deformities of the superior maxilla are very unusual. Several theories have been advanced to explain the mechanism of this change. It is probable, however, that no one cause is exclusively concerned in it, but that it is due to several factors which, combined, finally succeed in the production of the characteristic result. Among the explanations offered, that of David is one of the best. He believes that the deformity of the hard palate is caused by atmospheric pressure, the act of swallowing causing in persons suffering from nasal obstruction a partial vacuum in the upper pharynx and nasal cavities, as proved by the sunken condition of the tympanum of the ear, common in these cases. Rarefaction of the air above the hard palate with a continuation of the normal pressure below it tends to the constant pushing upward of the roof of the mouth. The younger the child, the more, presumably, will this influence be felt and the greater will be its effect. Again, according to some, the mouth-breathing habit compels the constant dropping of the lower jaw, which, hanging by the cheek from the superior maxilla, causes constant pressure upon the upper jaw. This produces flattening of the lateral alveolar arches and shortening of them, in consequence of which there is not sufficient space for the eruption of the canines when they are due, and they, therefore, grow forward.

According to Bazin, the deformity is still further exaggerated by the presence of the tongue in the floor of the mouth, which by its weight would tend to cause the lower jaw to expand beyond its normal limits, and thus still further impair the already imperfect coaptation of the lower teeth with those of the upper arch, while the absence of the tongue from the roof of the mouth would probably tend to increase the deformity of the hard palate itself.

Impaired development by reason of nasal obstruction of a part of the arch; the effect upon the arch of atmospheric pressure; the result of traction upon the outer walls of the maxillary region; and, finally, the influence of the tongue,—all these factors may contribute more or less to produce the deformity of the arch in question. It is my belief that of the causes above mentioned, atmospheric pressure and faulty development of the nasal region are probably the most important. With our present knowledge, it is impossible to determine with certainty which of them plays the most prominent part. Certain it is, however, that mouth-breathing itself, the efficient factor in the production of these secondary causes, is almost invariably attended with the deformities above described, as proved by innumerable cases, constantly seen by competent observers the world over. While, as before stated, brilliant results can be obtained in the correction of the faulty position of the teeth, the deformity of the hard palate is unfortunately irremediable. We have at present no method by which, when once established, it can be overcome. It is not necessary that the child should have attained considerable development in order that the deformity of the hard palate should be well established. On the contrary, it may exist at a very early age. I have seen it perfectly exhibited at fourteen months, while there is probably no reason why it should not occur even in younger children. It is highly important, therefore, that it should be, so far as possible, prevented from establishing itself, and this may best be done by the early recognition of the fact that the patient is a mouth-breather, and that some obstruction of the nose or pharynx is present. Such obstruction, when recognized, can always be removed, and, whether occasioned by adenoid hypertrophy, nasal obstruction from hypertrophic catarrh, or from enlarged faucial tonsils, should never be allowed to remain unrelieved. The operation for the removal of the offending tissue must vary somewhat with the nature, extent, and situation of the growth. As a rule, it is best performed under an anæsthetic, and to be thoroughly successful must be done with considerable care and skill.

The result of the removal of adenoids from the pharynx is most happy upon the whole well-being of the patient. In children the effect upon the development of the nose is sometimes very striking, and although the high arched condition never entirely disappears, the earlier the mouth-breathing habit is cured the better will be the opportunity for the palate to be relieved of the influences which are working against it, and for the deformity to decrease with the improved general development of the part. At present, removal of nasal and pharyngeal obstruction is the best and only preventive measure we have at hand.

May we not hope that the future may bring to us some efficient method by which these deformities, when once established, may also be overcome ?

### BIBLIOGRAPHY.

David, *Revue Mensuelle de Laryngologie*, 1883.

Sir Morrell Mackenzie, "Diseases of the Throat and Nose," vol. ii.

Henri Chatellier (Ballière et fils, Paris, 1890), "Maladies du Pharynx Nasal : Des Tumeurs Adenoides."

Scanes Spicer, *Transactions of the Odontological Society of Great Britain*, January, 1890, "On Nasal Obstruction and Mouth-Breathing as Factors in the Development of the Vaulted Palate and of Caries of the Teeth."

F. H. Hooper, *Medical and Surgical Reports of City Hospital of Boston*, Fourth Series, 1889, "The Mechanical Effects of Adenoid Vegetations in Children."

J. A. Bazin, *Dominion Dental Journal*, July, 1890, "Certain Peculiarities of the Maxillaries."

## Figures and Tables

**Fig. 1. f1:**
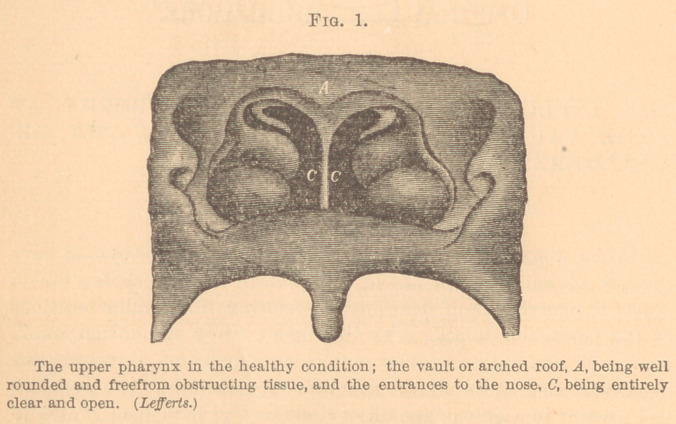


**Fig. 2. f2:**
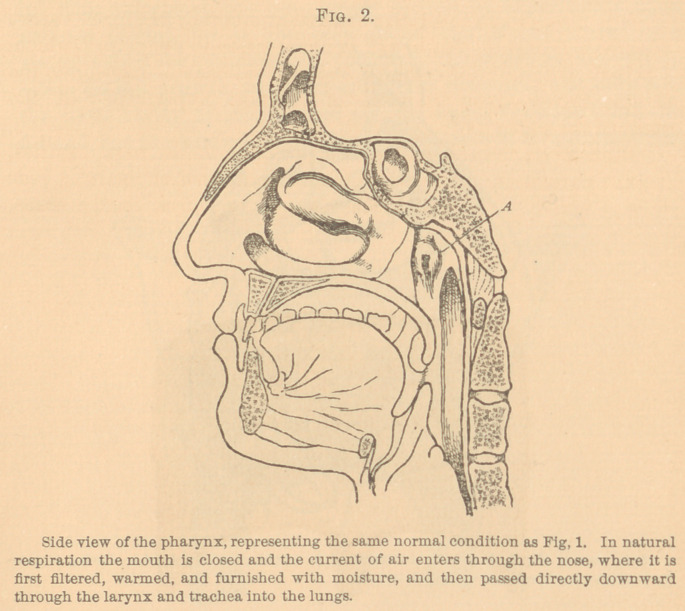


**Fig. 3. f3:**
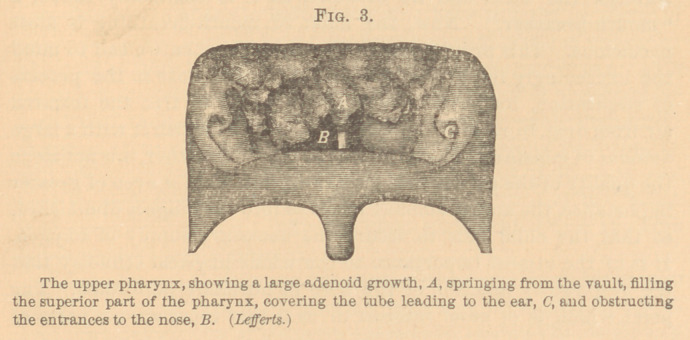


**Fig. 4. f4:**
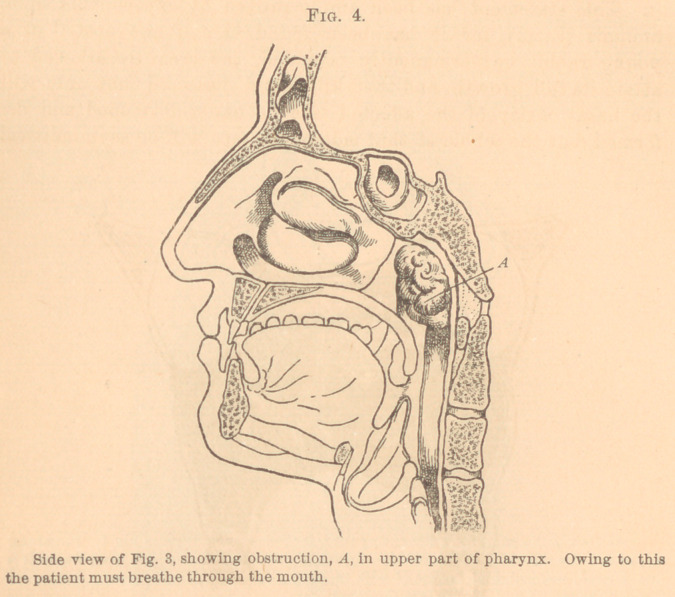


**Fig. 5. f5:**
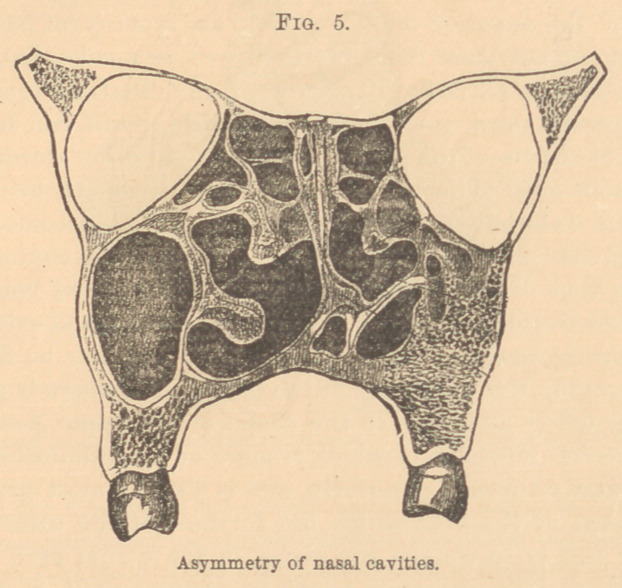


**Fig. 6. f6:**